# Cell Wall Invertase 3 Affects Cassava Productivity via Regulating Sugar Allocation From Source to Sink

**DOI:** 10.3389/fpls.2019.00541

**Published:** 2019-04-30

**Authors:** Wei Yan, Xiaoyun Wu, Yanan Li, Guanghua Liu, Zhanfei Cui, Tailing Jiang, Qiuxiang Ma, Lijuan Luo, Peng Zhang

**Affiliations:** ^1^Hainan Key Laboratory for Sustainable Utilization of Tropical Bioresources, Institute of Tropical Agriculture and Forestry, Hainan University, Haikou, China; ^2^National Key Laboratory of Plant Molecular Genetics, CAS Center for Excellence in Molecular Plant Sciences, Institute of Plant Physiology and Ecology, Shanghai Institutes for Biological Sciences, Chinese Academy of Sciences, Shanghai, China; ^3^Institute of Tropical and Subtropical Cash Crops, Yunnan Academy of Agricultural Sciences, Baoshan, China; ^4^University of Chinese Academy of Sciences, Beijing, China

**Keywords:** cassava, cell wall invertase, phloem loading, sugar allocation, storage root, productivity

## Abstract

Storage roots are the main sink for photo-assimilate accumulation and reflect cassava yield and productivity. Regulation of sugar partitioning from leaves to storage roots has not been elucidated. Cell wall invertases are involved in the hydrolysis of sugar during phloem unloading of vascular plants to control plant development and sink strength but have rarely been studied in root crops like cassava. *MeCWINV3* encodes a typical cell wall invertase in cassava and is mainly expressed in vascular bundles. The gene is highly expressed in leaves, especially mature leaves, in response to diurnal rhythm. When *MeCWINV3* was overexpressed in cassava, sugar export from leaves to storage roots was largely inhibited and sucrose hydrolysis in leaves was accelerated, leading to increased transient starch accumulation by blocking starch degradation and reduced overall plant growth. The progress of leaf senescence was promoted in the *MeCWINV3* over-expressed cassava plants with increased expression of senescence-related genes. Storage root development was also delayed because of dramatically reduced sugar allocation from leaves. As a result, the transcriptional expression of starch biosynthetic genes such as *small subunit ADP-glucose pyrophosphorylase*, *granule-bound starch synthase I*, and *starch branching enzyme I* was reduced in accordance with insufficient sugar supply in the storage roots of the transgenic plants. These results show that MeCWINV3 regulates sugar allocation from source to sink and maintains sugar balance in cassava, thus affecting yield of cassava storage roots.

## Introduction

Cassava (*Manihot esculenta* Crantz) is one of the most important food crops producing starch as a source of dietary calories and for industrial applications, especially in the tropics (De Souza et al., 2017). Its storage root is the main organ indicating cassava productivity by accumulating starch from CO_2_ fixation (Yang et al., 2011). Increasing cassava yield is needed due to continuous growth of the world population and global warming (Godfray et al., 2010; El-Sharkawy, 2014). Therefore, understanding the regulatory mechanism of photosynthate partitioning and its role in cassava production is critical for improving cassava yield through molecular breeding.

Photosynthate partitioning in cassava is a process of long-distance transport of sugars from aerial leaves to subterranean storage roots via the phloem vascular system. In vascular plants, sucrose is synthesized in leaves and then transported and loaded into phloem via symplastic or apoplastic pathways by sugar transporters (Chen et al., 2015; Khadilkar et al., 2016; Nieberl et al., 2017). During this process, invertases (INVs), a key enzyme that hydrolyzes sucrose to glucose and fructose, regulate carbon partitioning and sugar metabolism (Ruan et al., 2010). INVs can be distinguished according to subcellular localization and pH optima as acidic cell wall INV (CWINV), acidic vacuolar INV (VINV), and neutral to alkaline cytoplasmic INV (CINV) (Sturm, 1999). All INVs play key roles in plant growth for pollination, fruit ripening, and cellulose biosynthesis (Qin et al., 2016; Goetz et al., 2017; Rende et al., 2017). CWINV is located at the cell wall in apoplasts and is important for regulating both phloem loading and unloading of sucrose. Since the substrates and products of this kind of enzyme are both nutrients and signal molecules, CWINVs participate in many aspects of plant development and growth (Roitsch and Gonzalez, 2004). These include sucrose and starch accumulation in carrot roots (Tang et al., 1999) and tomato fruit (Zhang N. et al., 2015); pathogenesis in tomato (Schaarschmidt et al., 2006; Kocal et al., 2008; Bonfig et al., 2010), tobacco (Essmann et al., 2008), and rice (Sun et al., 2014); and seed development in tomato (Jin et al., 2009), cotton (Wang and Ruan, 2012; Wang et al., 2013), maize, sorghum, and rice (Chourey et al., 2010; Jain et al., 2010; Li et al., 2013; French et al., 2014). These CWINVs might perform sucrose hydrolysis in various tissues, mainly in phloem, to facilitate phloem loading/unloading. A recent study showed that the tomato CWINV LIN5 protein functions specifically in cell walls of sieve elements in ovaries immediately prior to anthesis and in young fruitlets with increased activity during ovary-to-fruit transition (Palmer et al., 2015). Heterologous expression of a yeast CWINV in plants altered sugar transport and impaired whole plant development due to disturbed assimilate partitioning (von Schaewen et al., 1990; Sonnewald et al., 1991; Heineke et al., 1994). Although the function and regulation of CWINV have been broadly studied in plant sink tissues (seed, fruit), the pivotal role of CWINV on sugar loading in source leaves has not been systematically studied and its effects on storage root development are unknown.

Because of its importance in sucrose metabolism and carbon partitioning in plants, regulation of INV is dynamic for CWINV, VINV, and CINV at both transcriptional and post-transcriptional levels (Huang et al., 2007). INV transcription can be increased by pathogen infection, mycorrhization, wounding, and hormonal treatments (Sturm and Chrispeels, 1990; Benhamou et al., 1991; Ehness and Roitsch, 1997; Hall and Williams, 2000; Blee and Anderson, 2002; Pan et al., 2005). Wall-associated kinase as well as other kinases including serine/threonine kinase, tyrosine protein kinase, and phosphatases may affect INV function with variable modes of regulation. More importantly, CWINV activity can be regulated by a family of proteinaceous inhibitors known as cell wall inhibitor of fructosidase (CIF, Rausch and Greiner, 2004), affecting downstream sugar signaling cascades (Balibrea et al., 2004; Jin et al., 2009). Their interaction was disclosed by a structural study using the 2.6 Å crystal structure of Arabidopsis cell-wall invertase INV1 in complex with a CIF from tobacco (Hothorn et al., 2010). This study determined that the extracellular sucrose concentration and pH changes regulate association of the complex. An early study confirmed that CWINV was strongly protected by sucrose against proteinaceous inhibitors (Sander et al., 1996). In tomato, increased CWINV activity following down-regulation of its inhibitor expression delayed leaf senescence and increased seed weight and fruit sugar level (Jin et al., 2009; Zhang N. et al., 2015). Constitutive expression of a tobacco CWINV inhibitor prevented cold-induced sweetening of potato tubers (Greiner et al., 1999). CWINV activity was also important in regulating the expression of ZmMRP-1, a key transcription factor involved in transfer cell layer differentiation of maize endosperm (Bergareche et al., 2018). Increased starch content up to 20% in the kernels of transgenic maize overexpressing CWINVs have been reported, but lacking further investigation of expression profiles related to starch biosynthetic enzymes (Li et al., 2013), such as ADP-glucose pyrophosphorylase (AGPase), granule-bound starch synthase (GBSS), and branching enzyme (BE).

In cassava, genome-wide identification, expression, and activity analysis of the INV gene family as well as INV inhibitor was reported recently (Geng et al., 2013; Yao et al., 2014, 2015). However, their function in regulating source-sink carbon partitioning and effects on plant phenotype development in cassava have not been studied. To elucidate regulation of carbon partitioning on cassava storage root development, in this study the CWINV gene *MeCWINV3* was investigated in transgenic cassava using constitutive over-expression. The results showed that MeCWINV3 is important for sugar export in phloem loading, thus influencing carbon partitioning, storage root starch accumulation, and yield of cassava. MeCWINV3 may be involved in the creation of a weak gradient between the place of sucrose production (mesophyll) and the place of phloem loading (minor veins) in source leaves. Overexpression of MeCWINV3 leads to reduced sugar allocation from source to sink. Consequently, starch synthesis in storage roots was reduced and progress of leaf senescence was promoted.

## Materials and Methods

### Gene Cloning and Phylogenetic Analysis

The *MeCWINV3* gene (GenBank accession No. JN801147.1) was cloned from cDNA of cassava cv. TMS60444 according to sequence information from NCBI. To analyze its phylogenetic relationship with other INVs, a phylogenetic tree of 12 INV amino acid sequences from Arabidopsis (*Arabidopsis thaliana*) and cassava (*Manihot esculenta*) obtained from NCBI GenBank were aligned using maximum likelihood implemented in MEGA6 (Tamura et al., 2013). Bootstrap values were obtained with 1000 replications. Homology analysis was also performed by BLASTing cassava MeCWINV3 amino acid sequence with other CWINVs from *A. thaliana*, *Bambusa oldhamii*, *Malus domestica*, and *Oryza sativa*.

### Vector Construction, Plant Transformation, and Growth Conditions

The full length *MeCWINV3* gene was cloned and inserted into the binary vector pCAMBIA1301s controlled by the CaMV 35S promoter. The plasmid, harboring the hygromycin phosphotransferase gene (*hpt*) for selection of transgenic plants, was introduced into *Agrobacterium tumefaciens* strain LBA4404 by electroporation. Transformation of cassava was performed using a friable embryogenic suspension of cv. TMS60444 and transgenic plants were regenerated from hygromycin containing regeneration medium as described previously (Zhang et al., 2000). After root screening under selection, the putative transgenic cassava lines were macro-propagated on shoot culturing medium. Three to four weeks old plantlets of wild type (WT) and transgenic lines were planted in pots with nutrient soil (mixture of soil, perlite, and vermiculate, 1:1:1) and grown in the greenhouse (16 h/8 h of light/dark, 30°C/22°C day/night). Two months old potted plants were used for physiological analysis and ^13^C labeling assay. Stem cuttings from greenhouse-grown plants were collected and cultivated in the field at the Wushe Plantation for Transgenic Crops, Shanghai (31°13948.0099N, 121°28912.0099E) in early May. After 6 months of growth, the plants were harvested for phenotype evaluation in early November.

### Sucrose and Drought Treatment

Four months old cassava seedlings from *in vitro* cultures were used for hydrophobic culturing with a liquid medium as described by Fan et al. (2017). Supplementation of 30 or 60 mM sucrose was used for sugar treatments and leaves were collected for RNA extraction and gene expression analysis.

Pot-grown cassava plants under similar growth conditions for 2 months in the greenhouse were used for drought treatment. The drought experiment was carried out in a greenhouse environment by water depletion and phenotypes were observed with regular photography. After 35 days of drought treatment, the phenotypes of plants were observed.

### Subcellular Location and Expression Pattern Assay

The full length *MeCWINV3* gene was subcloned into vector PA7-GFP for the localization study. The constructed plasmid was embedded using gold powder and introduced into onion epidermis with a helium-driven particle gun (Bio-Rad PDS 1000/He). The PA7-GFP empty vector was used as a control. After 24 h of expression, the transformed onion epidermis was observed under 488 nm excitation fluorescence using an Olympus FV1000 confocal microscopy (Olympus, Tokyo, Japan).

The upstream sequence of *MeCWINV3* (1,890 bp in length) was obtained from the cassava genome database (*Manihot esculenta v6.1*)^1^ and cloned from genomic DNA of cultivar TMS60444. The promoter fragment was inserted into vector pCAMBIA1300-GUS between the SacI and XhoI sites. Then the GUS-expressing plasmid was introduced into *A. tumefaciens* strain GV3101 and used for transformation of *A. thaliana* ecotype Col-0. Transformed Arabidopsis was screened on 1/2 MS with 30 mg/l hygromycin B. The positive homozygotes were stained with GUS solution overnight and decolorized with 75% ethanol for photography.

### DNA and RNA Extraction, Southern Blotting, and qRT-PCR

Genomic DNA from leaves was isolated from wild-type and transgenic cassava plantlets as described by Xu et al. (2010). To perform Southern blot analysis, about 20–30 μg genomic DNA was digested by EcoRI and then separated using a 0.8% agarose gel. After electrophoresis, DNA was transferred onto a positively charged nylon membrane (Roche Diagnostics, Mannheim, Germany). Integrated T-DNA was detected after DNA hybridization using a digoxigenin labeled *hpt* probe which was synthesized with a PCR DIG Probe Synthesis Kit (Roche). The procedure of hybridization and detection was followed according to the instructions of the DIG-High Prime DNA Labeling and Detection Starter Kit II (Roche).

Total mRNA was extracted from cassava samples using RNAplant Plus Reagent and RNAprep Pure Plant Kit (TIANGEN, Beijing, China). cDNA was synthesized by reverse transcription of 2 μg total mRNA with ReverTra Ace qPCR RT Mix (TOYOBO, Osaka, Japan). qRT-PCR was performed using THUNDERBIRD SYBR qPCR Mix (TOYOBO) in a Bio-Rad CFX96 thermocycler as follows: 1 min pre-incubation step at 95°C, 40 cycles of 95°C for 15 s, 60°C for 15 s, and 72°C for 20 s. The expressions of *MeCWINV3* and its orthologs *MeCWINV1* to *MeCWINV6* were analyzed according to corresponding experimental designs, including tissue specificity and responses to nycthemeral cycle and sucrose. To detect the effects of *MeCWINV3* overexpression in cassava, the expressional changes of sucrose transporter genes *MeSUT1*, *MeSUT2*, and *MeSUT4*, and starch biosynthetic genes *MeAPS* (*small subunit ADP-glucose pyrophosphorylase*), *MeGBSSI* (*granule-bound starch synthase I*), and *MeSBEI* (*starch branching enzyme I*) were analyzed in leaves or storage roots of cassava plants. The expressions of senescence-related genes were also carried out using mature leaves. These genes include *NAC83* (*NAC domain-containing protein 83-like*, XM_021768763.1), *SAG12* (*senescence-specific cysteine protease SAG12-like*, XM_021749212.1), *Osl* (*gamma-aminobutyrate transaminase 3*, XM_021765427.1), and *Osh* (*alanine–glyoxylate aminotransferase 2*, XM_021768525.1). The gene expression data were normalized by cassava β-actin gene. Ct method was used to calculate relative expression level as follows: ΔCt = Ct (target gene)-Ct (beta-actin) ΔΔCt = ΔCt (sample)- ΔCt (reference gene). Samples with lowest expression level or WT were defined as reference genes (Schmittgen and Livak, 2008). Primer pairs used for PCR analysis were provided in Supplementary Table S1.

### INV Enzymatic Activity Measurement

Leaves and storage roots of three independent 5 months old plants per line were harvested from the field. The same types of samples were mixed and smashed in liquid nitrogen. Each sample (0.2 g) was mixed with 1 ml extraction buffer containing 100 mM HEPES-KOH (pH 7.4), 5 mM MgCl_2_, 1 mM EDTA, 1 mM EGTA, 1 mM PMSF, 5 mM DTT, 1 ml/l Triton X-100, 200 ml/l glycerol, and 5 mM thiourea, vortexed for 3 min and centrifuged at 4°C, 12,000 rpm for 5 min. Extraction buffer (0.5 ml) was added to the pellet to extract again as above and then extracts were mixed with the supernatant for CINV and VINV activity analysis. The final pellet was resuspended with 1.5 ml extraction buffer and vortexed thoroughly for CWINV activity analysis. All WT and transgenic lines had three replicates.

The INV activity assay was performed as described previously (Tomlinson et al., 2004) with some modifications. To measure INV activity, 40 μl extract was added into 360 μl reaction buffer (for CINV: 0.1 M sucrose, 50 mM Bicine-KOH, pH 7.6; for VINV: 0.1 M sucrose, 50 mM NaAc, pH 4.3; for CWIN: 0.1 M sucrose, 50 mM NaAc, pH 4.7) and incubated at 30°C for 1 h and then treatment at 85°C for 5 min was used to inactivate enzyme activity. After adding 400 μl chloroform for sugar extraction, the reaction mixture was vortexed and then centrifuged at 12,000 rpm for 5 min. The purified supernatant was kept as reaction solution for the next step. For VINV and CWINV analysis, 60 μl Tris-HCl (1 M, pH 8.0) was added. Finally, 760 μl glucose reaction buffer (100 mM HEPES-KOH pH 7.4, 2.25 mM MgCl_2_, 1.1 mM ATP, 1.1 mM NADP, 0.2 U hexokinase, and 0.2 U NADP-dependent G6P dehydrogenase) was added into the above reaction solution and incubated at 25°C for 1 h. The absorbance was measured at 340 nm using a Beckman Coulter DU730 Nucleic Acid/Protein Analyzer.

### ^13^C-Labeling and Phloem Exudate Collection

To measure phloem loading capacity, the ^13^CO_2_ labeling method was used for a phloem loading assay as described previously (Gui et al., 2014) with some modifications. In brief, 2 months old cassava plants from the greenhouse were labeled with one leaf in a sealed glass box. ^13^CO_2_ was released by the reaction between Na_2_^[13]^CO_3_ as well as an overdose of hydrochloric acid through a syringe. After 12 h of photosynthesis, the plant experienced a 4 h chase under dark. Leaf, phloem exudate, and root samples were then collected. Total sugar was extracted by the method described below and analyzed using GC-MS with same instruments and conditions described previously (Gui et al., 2014). Briefly, after derivatization by N,N-dimethylformamide (Sigma-Aldrich) containing 0.1% pyridine (Sigma-Aldrich) and N, O-bis-(trimethylsilyl)-trifluoroacetamide (Sigma-Aldrich), samples were injected into an Agilent 6890 instrument with an Agilent 5975 inert mass selective detector and an Agilent HP-5MS column (30 mm × 0.25 mm × 0.25 μm film thickness). Helium was used as the carrier gas at 1.5 mL/min, and detector temperatures were set to 310°C with electron ionization in positive mode. The profile was as follows: holding for 5 min at an initial temperature of 120°C, gradually increasing the temperature to reach 270°C at 4°C/min and ramping up the temperature at 20°C/min to a final temperature of 310°C. Data were acquired and processed with an Agilent ChemStation system.

### Water-Soluble Carbohydrates and Starch Assays

Phloem exudate was collected as described previously (King and Zeevaart, 1974) with some modifications. Briefly, 2 months old plants from the greenhouse were cut rapidly at the stem 2 cm from the base using a clean knife blade, rinsed with distilled water, and put into 20 mM EDTA solution for 2 h in the dark at 30°C and 95% humidity. Then the plant cuttings were transferred into ddH_2_O for 5 h and the samples were finally filtered for HPLC analysis of glucose, fructose, and sucrose. The Agilent technologies HPLC column (ZORBAX Carbohydrate column; 4.6 × 150 mm, 5 μm) with a differential refraction detector was used for sugar analysis. The mobile phase consisted of 75% acetonitrile with a flow rate of 0.8 ml/min and the temperature of the column was held at 35°C. Sugars were identified by retention time of the standards and sugar concentration of samples were calculated from the external standard curve.

Leaves collected from the field at 6 a.m. were decolored by 75% ethanol overnight and stained with 1% Lugol’s iodine solution (3.75 g KI and 1.25 g I_2_ diluted in 500 ml H_2_O) for 4 h at room temperature. Excess Lugol’s iodine solution was removed using distilled water. The treated leaves were photographed with a Nikon D7000 digital camera.

Leaves and storage roots of three independent 5 months old plants per line were harvested from the field. The same types of samples were mixed and smashed in liquid nitrogen. Frozen samples (100 mg) were dissolved in 0.7 ml 80% ethanol, thoroughly blended, and incubated at 70°C for 2 h. Then 0.7 ml ddH_2_O was added to each sample and the samples were vortexed and centrifuged at 12,000 rpm for 10 min. The supernatant was purified twice using chloroform to remove chlorophyll and transferred to glass bottles for HPLC analysis of glucose, fructose, and sucrose. Conditions of HPLC analysis were according to Zhou et al. (2017). The pellet was washed three times with 80% ethanol for starch content analysis. Total starch content was analyzed using a total starch kit (Megazyme, Wicklow, Ireland).

### Superoxide Dismutase and Catalase Activity

Cassava leaves (1 g) were mixed with 5 ml enzyme buffer (50 mmol/l phosphate buffer, 1% PVP, and 1 mmol/l EDTA), ground in an ice bath and centrifuged for 15 min with 12,000 rpm at 4°C. The supernatant was collected and kept on ice. The buffer solution of 1.8 ml enzyme extraction, 0.3 ml 130 mmol/l methionine solution, 0.3 ml 750 mol/l NBT solution, 0.3 ml 20 mol/l riboflavin, and 0.3 ml supernatant was added into the tube. Buffer was added to the control tube instead of the enzyme solution. A black cardboard cover was used to shade the light and all tubes were placed under a lamp for 10–20 min. When the control tube turned blue and the sample tube remained yellow, the reaction was complete and the absorbance of each tube at 560 nm was measured. Superoxide dismutase (SOD) activities were calculated using the following equation: SOD activity = (OD_0_-OD_S_) × VT/OD_0_ × 0.5 × FW × V1; where OD_0_: control absorption; OD_S_: sample absorption; VT: total sample volume; V1: sample volume used; FW: fresh weight.

The reaction system included 1.6 ml enzyme buffer (50 mmol/l phosphate buffer, 1% PVP, and 1 mmol/l EDTA), 0.2 ml supernatant, and 0.2 ml 0.1 mol/l H_2_O_2_. Immediately after adding H_2_O_2_, the tube was mixed upside down, and the absorbance was measured at 240 nm. The absorbance was read every 1 min for 5 min. The catalase (CAT) enzyme activity unit was calculated by reducing the absorbance of 240 nm by 0.1 in 1 min.

### Statistical Analysis

Data from at least three independent replicates were presented as means ± SD. Analysis of variance (ANOVA) by Duncan’s multiple range tests conducting all pairwise multiple comparison procedures was performed using SigmaPlot software, version 12.5 (Systat, San Jose, CA, United States). A value of *p* < 0.05 was considered a significant difference, which are represented by different letters.

## Results

### *MeCWINV3* Encodes a Typical CWINV That Is Mainly Expressed in Leaf Vascular Bundles and Is Inducible by Sucrose

The *MeCWINV3* gene was 1,731 bp in length and encoded a protein of 577 amino acids with a calculated molecular mass of 44.26 KDa and an isoelectric point of 6.477. MeCWINV3 (GenBank accession No. AFA46812.1) has 13 regions that are conserved in known acid INVs (Ji et al., 2005), including three conserved sequence domains – NDPNG (β-fructosidase motif), RDP, and WECP(V)D – and the predicted conserved glycosylation sites (Supplementary Figure S1A; Yao et al., 2014). Amino acid sequence alignment showed that MeCWINV3 shared at least 71.26% identity with CWINs from *A. thaliana*, *Bambusa oldhamii*, *Malus domestica*, and *Oryza sativa* (Supplementary Figure S1A). The phylogenetic tree constructed using all cassava and Arabidopsis CWINV genes showed that MeCWINV3 is closely related to AtcwINV1, AtcwINV3, and AtcwINV5, which belong to group III of CWINs in Arabidopsis (Yao et al., 2014; Supplementary Figure S1B). Moreover, MeCWINV3 is predicted to be localized in the cell wall by WoLF PSORT (Horton et al., 2007).

**FIGURE 1:**
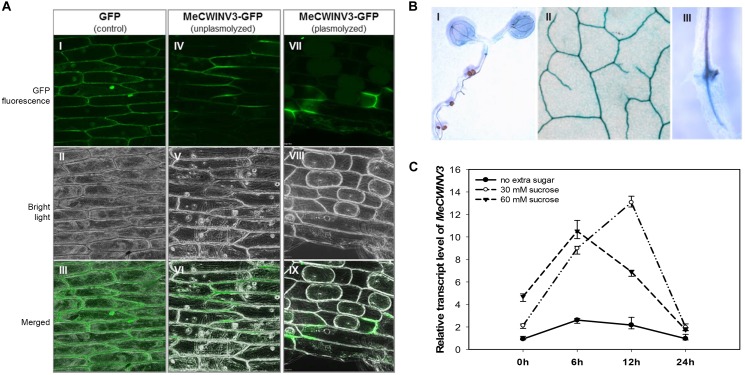
Localization and expression pattern of MeCWINV3. **(A)** MeCWINV3-GFP fusion showed cell wall localization in onion epidermis cells (I–III, GFP control; IV–VI, unplasmolyzed; VII–IX, plasmolyzed). **(B)** GUS assay showing MeCWINV3 promoter activity in the vascular bundle of transgenic Arabidopsis, (I, seedling; II, leaf; III, root). **(C)** Transcriptional response of *MeCWINV3* in hydroponic seedlings under 30 and 60 mM sucrose treatments in cassava plants.

Transient expression of the MeCWINV3-GFP fusion protein showed that MeCWINV3 was located in the cell wall of the onion epidermis cells, as indicated by GFP fluorescence, under unplasmolyzed and plasmolyzed conditions. For the control, GFP fluorescence was observed ubiquitously in the cell membrane, cell wall, and nucleus (Figure 1A). GUS staining of *MeWICNV3* promoter::GUS transgenic Arabidopsis plants showed that *MeCWINV3* was expressed mainly in vascular bundles of the whole seedling, including young and mature leaves, hypocotyl, and roots (Figure 1B).

The transcription of *MeCWINV3* was also inducible in cassava plants treated with 30 mM or 60 mM sucrose using the hydroponic seedling culturing system. In a 24 h regime, the expression of *MeCWINV3* increased fourfold with minimum expression at 6 h; the highest expression was detected at 12 h under 30 mM sucrose treatment (Figure 1C).

### *MeCWINV3* Responds to a Day/Night Cycle and Is Predominantly Expressed in Leaves

**FIGURE 2:**
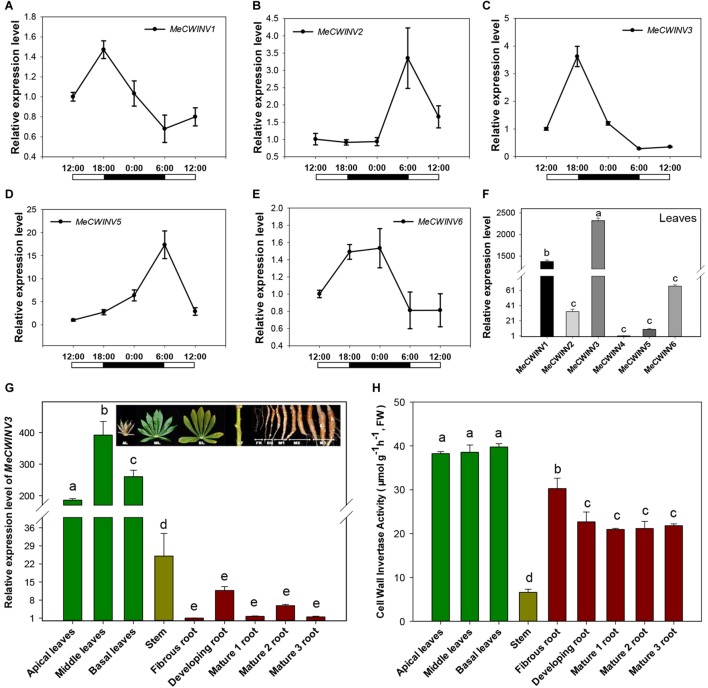
Expression patterns of CWINVs in cassava. **(A–E)** Relative expression levels of five CWINV genes in cassava leaves under a day/night cycle. **(F)** Relative transcript levels of six cassava CWINV genes in cassava leaves collected from pot-grown plants at 10:00 a.m. **(G,H)** Relative expression levels of *MeCWINV3* **(G)** and CWINV activities **(H)** in different types of cassava organs including leaves, stem, and roots. Data represent means ± SD of three independent assays. Different letters indicate significant differences (one-way ANOVA, *p* < 0.05).

Transcriptional analysis of cassava CWINs showed a differential expression pattern in response to day/night cycle. *MeCWINV3* had a similar expression pattern to *MeCWINV1*, with the highest transcript level at the end of the light period (18:00) and lowest at the end of the dark period (6:00) (Figure 2A,C). Meanwhile, *MeCWINV2* and *MeCWINV5* had different trends of peak expression at the end of the dark period (6:00) and with minimum expression at noon (12:00) (Figure 2B,D). The expression of *MeCWINV6* showed higher expression during the dark period and lower expression during the light period (Figure 2E). The differential expression patterns of these INVs might reflect diverse functions of INVs in cassava to respond to a nycthemeral cycle for sugar partitioning. Of the INVs studied, *MeCWINV1* and *MeCWINV3* are predominantly expressed in leaves and *MeCWINV3* had the highest expression (Figure 2F).

The transcript levels of *MeCWINV3* in cassava plant varied in different organs, i.e., leaves, stems, and roots based on real-time RT-PCR analysis. The highest transcript level was observed in leaves and the lowest was in roots (Figure 2G). The differences between leaves and roots were about 200-fold at minimum and 400-fold at maximum. In leaves, the highest expression level was observed in the middle leaves while the apical leaves had the lowest expression. Compared to leaves, very low expression was detected in the roots; among roots, storage roots including developing and mature storage root had relatively higher expression than the fibrous roots. During storage root growth, variation in *MeCWINV3* expression was also noticeable: it increased in the developing storage roots and then decreased in the mature storage roots at stage 1 (M1) to almost the same level as the fibrous root before rising to a higher level in the mature storage roots at stage 2 (M2); finally, expression was reduced again in the mature storage roots at stage 3 (M3), which is also the stage for harvest. Despite these observed changes, there were no significant differences in expression among different types of roots (Figure 2G).

Enzymatic activity of total CWINV activity in different cassava organs was also analyzed. All types of leaves had similar levels, about 38 μmol⋅g^-1^⋅h^-1^ (fresh weight). In roots, the highest activity (30.3 μmol⋅g^-1^⋅h^-1^, fresh weight) was detected in fibrous root, about 20% higher than other types of storage root. There were no significant differences among the developing and mature storage roots. The lowest activity was found in the stem, accounting for less than one fifth of the expression in leaves (Figure 2H).

### Overexpression of *MeCWINV3* in Cassava Leads to Reduced Biomass Production and Storage Root Development

**FIGURE 3:**
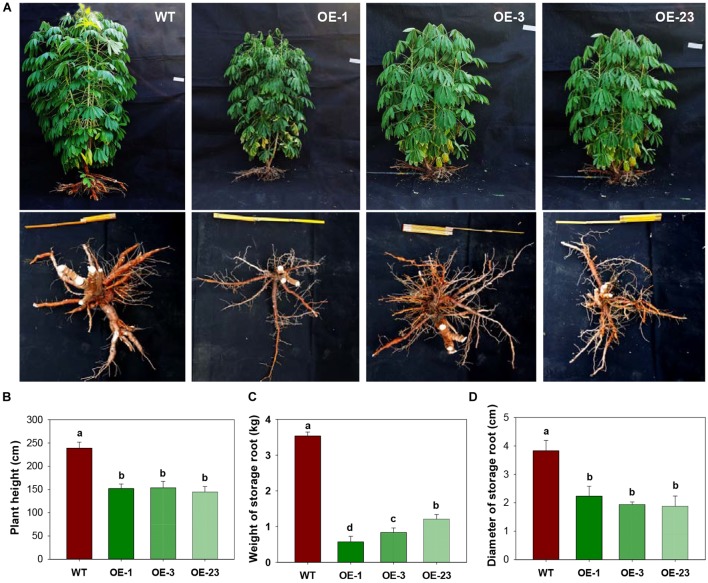
Field performances of transgenic cassava plants overexpressing *MeCWINV3*. **(A)** Whole-plant phenotype of wild-type (WT) and transgenic (OE lines) cassava plants. **(B–D)** Plant height **(B)**, storage root yield and **(C)**, and storage root diameter **(D)** of WT and transgenic plants. Different letters indicate significant differences (one-way ANOVA, *p* < 0.05).

Three transgenic cassava plant lines that had a single T-DNA integration (Supplementary Figure S2) were selected for molecular and physiological analyses. Compared with the wild-type cassava plants, the transgenic plants overexpressing *MeCWINV3* grew much more slowly with reduced size of aboveground parts during all stages (Figure 3A). The plant height of OE lines was significantly shorter than that of WT. At harvest, the transgenic plants were 35.7–39.5% shorter than WT (Figure 3B). Significantly reduced storage root size was also found in the transgenic lines. Total fresh weight of storage roots per plant in transgenic lines was 65.8–83.8% lower than that of WT (Figure 3C). The diameter of storage roots in transgenic lines was 42.1–50.6% thinner than that of WT (Figure 3D). The reduced root size led to altered phenotype with reduced yield in OE transgenic lines.

### *MeCWINV3* Overexpression in Cassava Alters Carbon Export and Allocation in Leaves

**FIGURE 4:**
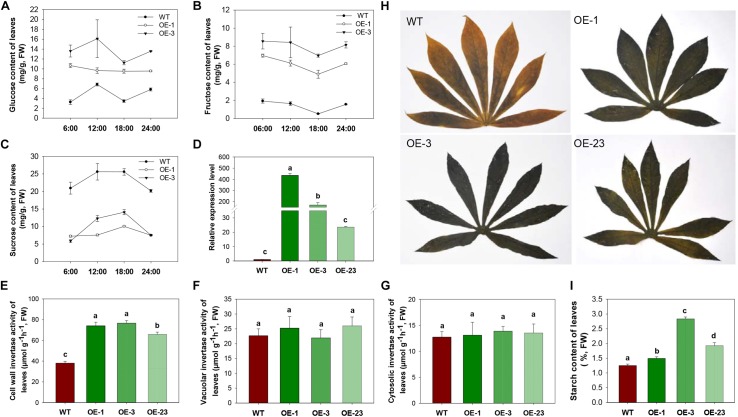
INV activities, sugar contents and starch accumulation in leaves of wild-type (WT) and the *MeCWINV3-*overexpressed (OE) cassava plants. **(A–C)** Contents of glucose **(A)**, fructose **(B)**, and sucrose **(C)** under a day/night cycle. **(D)** Relative transcription level of *MeCWINV3*. **(E–G)** The enzymatic activity assay of CWINV **(E)**, VINV **(F)**, and CINV **(G)**. **(H,I)** Iodine staining of leaves collected at 6:00 a.m. **(H)** and their total starch contents **(I)**. Different letters indicate significant differences (one-way ANOVA, *p* < 0.05).

To study the influence of CWINV on carbon allocation, sugar and starch content of leaves and storage roots were assessed. During day and night, glucose and fructose content in leaves were always higher in the OE lines, while sucrose content dramatically decreased in the transgenic lines compared to WT (Figure 4A–C). Both the transcript level of *MeCWINV3* and total enzyme activity of CWINVs were higher in leaves of the transgenic lines than in WT (Figure 4D,E). The activities of other types of INV, i.e., VINV and CINV, in the leaves of transgenic lines were not obviously different from WT (Figure 4F,G). Further transcription analysis of other CWINV isoform genes showed that expressions of *MeCWINV1*, *MeCWINV2*, and *MeCWINV4* were significantly increased by 16.6-, 4.8-, and 10.5-times, on average, in the leaves of the OE lines, respectively. The transcriptions of *MeCWINV5* and *MeCWINV6* were less affected (Supplementary Figure S4). Iodine staining of leaves collected at 6 a.m. showed that the transgenic cassava plants accumulated much more starch than WT, showing a darker blue indicating the iodine-starch complex (Figure 4H). Total starch analysis also demonstrated that starch content of the leaves of OE lines was significantly higher than that of WT (Figure 4I). Quantitative analysis of effective PSII quantum yield and non-photochemical quenching parameter showed no significant changes in photosynthetic performance of the transgenic leaves (Supplementary Figure S3).

**FIGURE 5:**
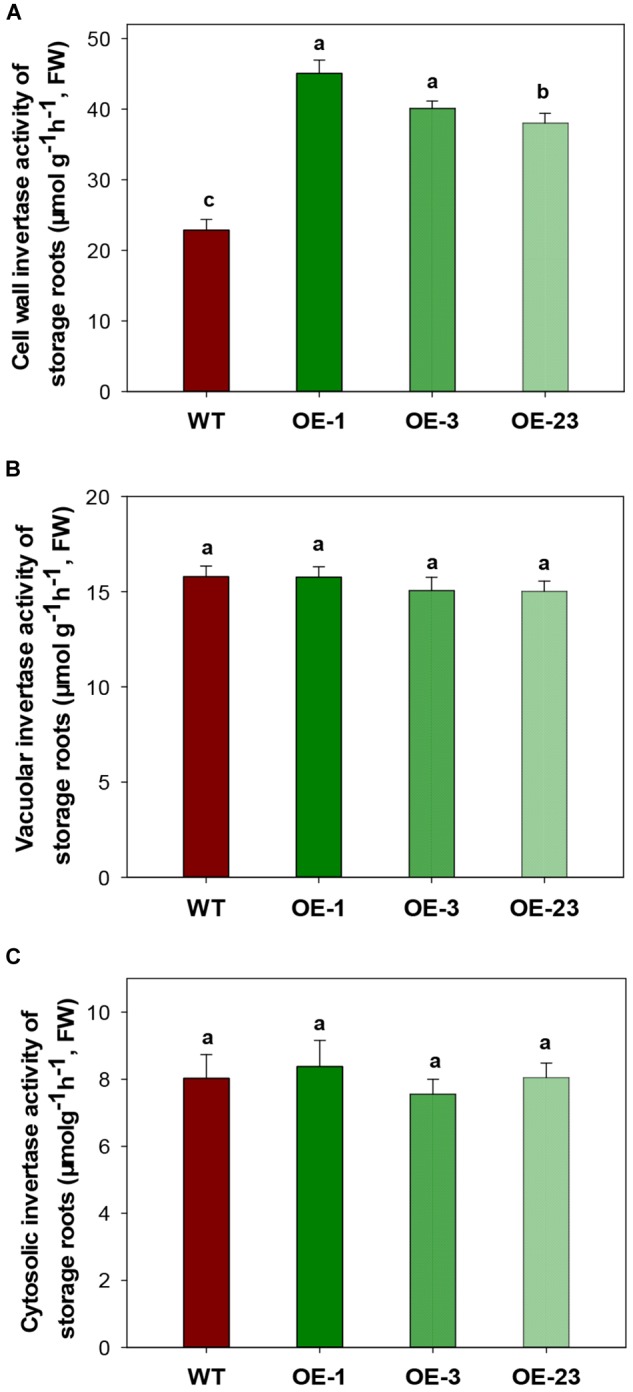
INV activity assay in storage roots of wild-type (WT) and the *MeCWINV3*-overexpressed (OE) cassava plants. **(A)** CWINV. **(B)** VINV. **(C)** CINV. Different letters indicate significant differences (one-way ANOVA, *p* < 0.05).

**FIGURE 6:**
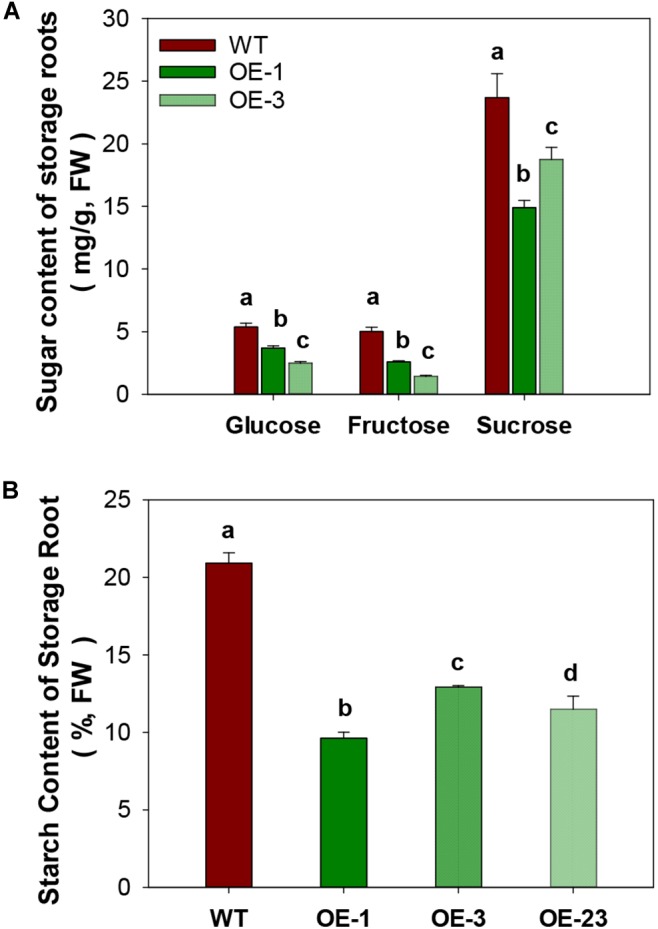
Total sugar and starch content in storage roots of wild-type (WT) and the *MeCWINV3*-overexpressed (OE) cassava plants. **(A)** Contents of glucose, fructose, and sucrose. **(B)** Starch contents. Different letters indicate significant differences (one-way ANOVA, *p* < 0.05).

CWINV activities were also significantly higher (about 2 times) in storage roots of the OE lines (Figure 5A) but this was not the case for either vacuole or cytosolic INVs (Figure 5B,C). The over-expression of *MeCWINV3* in OE transgenic lines is attributed to the increase in total CWINV activity. However, sugar content, including glucose, fructose, and sucrose, was significantly reduced in storage roots of transgenic plants (Figure 6A). Moreover, the starch content in storage roots decreased by 38.2–54% compared to that of WT (Figure 6B). No obviously change was detected in the expression of *MeSUT1* which is responsible of long-distance sucrose transport in phloem loading (Supplementary Figure S5). These results indicated limited carbon transport from source to sink.

**FIGURE 7:**
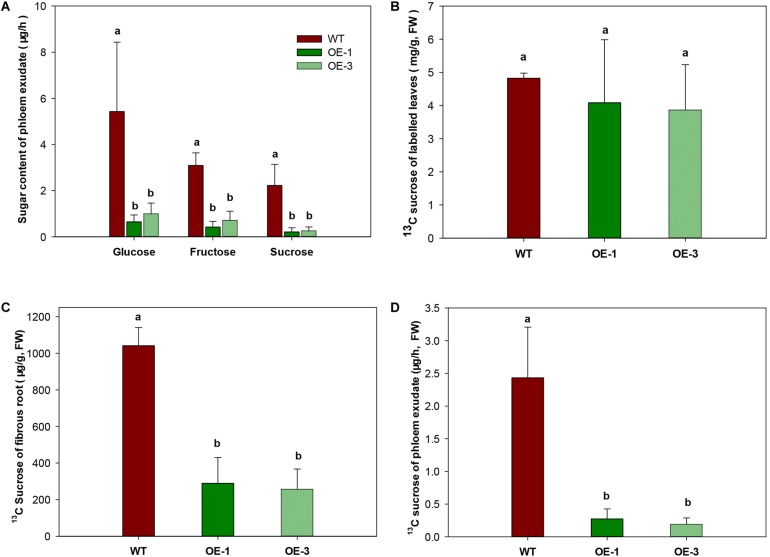
Sugar transport capacity in wild-type (WT) and the *MeCWINV3*-overexpressed (OE) cassava plants. **(A)** Contents of glucose, fructose, and sucrose in phloem exudates. **(B)** Content of ^13^C-labeled sucrose in leaves of ^13^CO_2_-fed plants. **(C)** Content of ^13^C-labeled sucrose in fibrous roots of ^13^CO_2_-fed plants. **(D)** Content of ^13^C-labeled sucrose in phloem exudates of ^13^CO_2_-fed plants. Different letters indicate significant differences (one-way ANOVA, *p* < 0.05).

To investigate whether the export of sugar was affected by increased CWINV activity, phloem exudate was analyzed in WT and transgenic plants fed with the ^13^C isotope labeled CO_2_. Sugar in phloem exudate was significantly different between the OE transgenic plants and WT. Compared to WT, glucose and fructose decreased at least 77.3% and sucrose decreased 88.8% in the OE transgenic lines (Figure 7A). Further analysis of ^13^C labeled sucrose, which is the main exporting form of photosynthesis assimilates, by GC-MS showed that no difference appeared among two transgenic lines and WT in leaves (Figure 7B), but in fibrous roots ^13^C sucrose was significantly reduced (Figure 7C). About 70–85% less phloem exudate was detected in OE transgenic plants compared to WT (Figure 7C,D), which was consistent with above result (Figure 7A).

### Overexpression of *MeCWINV3* in Cassava Reduces Starch Biosynthesis in Storage Roots

**FIGURE 8:**
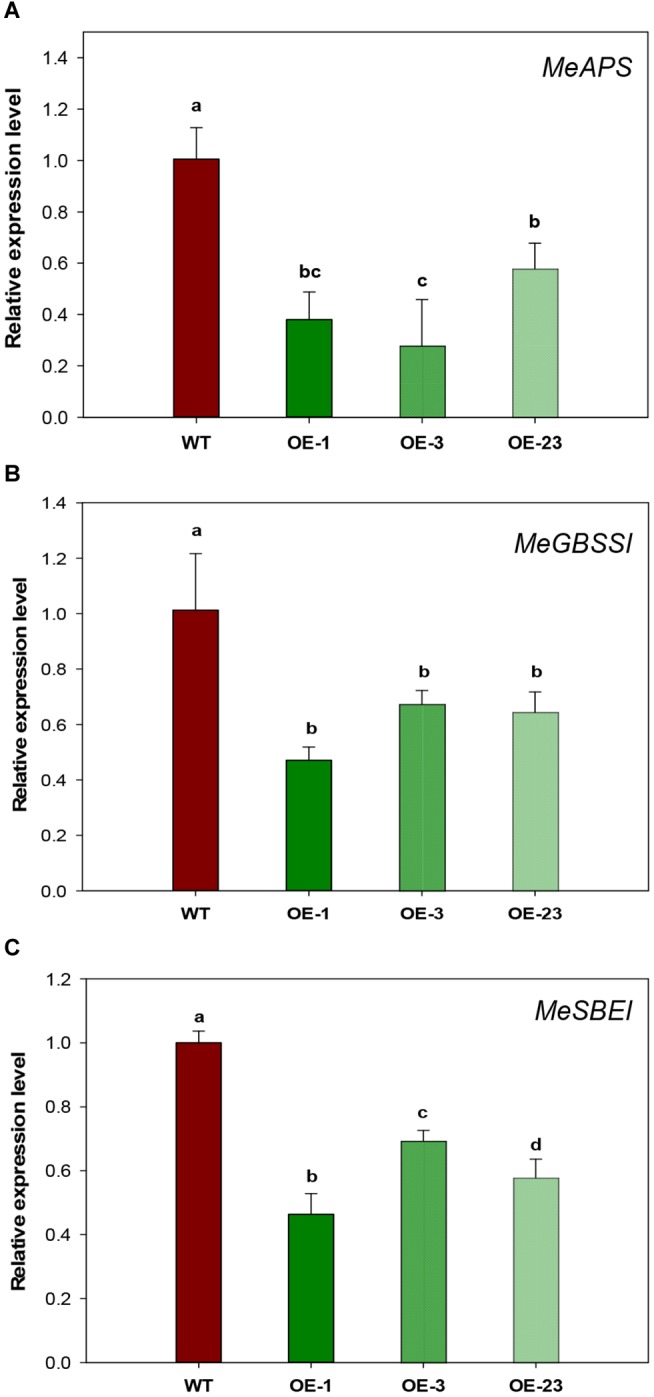
Relative transcription levels of starch biosynthetic genes in storage roots of wild-type (WT) and the *MeCWINV3*-overexpressed (OE) cassava plants. **(A)** *MeAPS*, the small subunit ADP-glucose pyrophosphorylase gene. **(B)** *MeGBSSI*, the granule-bound starch synthase I gene **(C)** *MeSBEI*, the starch branching enzyme I gene. Different letters indicate significant differences (one-way ANOVA, *p* < 0.05).

Since both sugar and starch contents were significantly decreased in the storage roots of OE transgenic cassava plants (Figure 6), starch biosynthetic genes were analyzed to check whether the reduced starch content was due to decreased starch biosynthesis. Significantly down-regulated expression was detected for three genes, *small subunit ADP-glucose pyrophosphorylase* (*APS*), *granule-bound starch synthase I* (*GBSSI)*, and *starch branching enzyme I* (*SBEI*), in the OE lines by qRT-PCR (Figure 8A–C). The reduction ranged from 43 to 72% for *MeAPS*, 40–50% for *MeGBSSI*, and 31–52% for *MeSBEI* among three OE transgenic lines.

### *MeCWINV3*-Overexpressing Cassava Plants Show Early Leaf Senescence and Increased Drought Sensitivity

**FIGURE 9:**
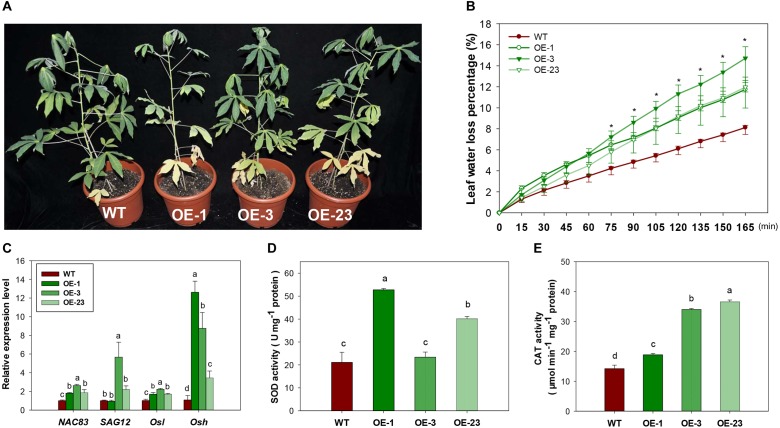
Leaf senescence phenotype, physiological changes and gene expression in the wild-type (WT) and *MeCWINV3*-overexpressed (OE) cassava plants. **(A)** Plant phenotype of early basal leaf senescence. **(B)** Transcript levels of senescence-related genes *NAC83* (NAC domain-containing protein 83-like, XM_021768763.1), *SAG12* (senescence-specific cysteine protease SAG12-like, XM_021749212.1), *Osl* (gamma-aminobutyrate transaminase 3, XM_021765427.1), and *Osh* (alanine–glyoxylate aminotransferase 2, XM_021768525.1). **(C)** Water loss capacity. **(D,E)** Total SOD and CAT activities in mature leaves. Different letters indicate significant differences (one-way ANOVA, *p* < 0.05).

Under normal growth conditions, an early leaf senescence phenotype was observed in the basal leaves of OE transgenic cassava lines compared to WT (Figure 9A). Leaves from transgenic lines had higher water loss (Figure 9B) and reduced H_2_O_2_ content (Supplementary Figure S6). Several genes related to the regulation of leaf senescence showed upregulated expression, especially the *Osh* gene (alanine-glyoxylate aminotransferase 2, XM_021768525.1, Figure 9C), indicating that the altered sucrose homeostasis affected senescence progress. Increased reactive oxygen species (ROS) generation may lead to the progression of senescence as indicated by increased enzymatic activity of SOD and CAT (Figure 9D,E).

**FIGURE 10:**
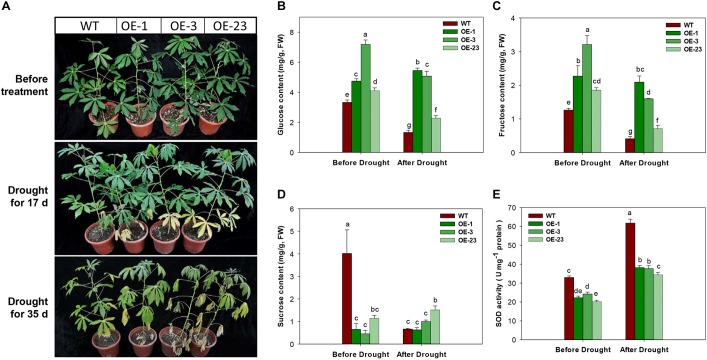
Phenotypical and physiological performance of wild-type (WT) and *MeCWINV3*-overexpressed (OE) cassava plants under drought treatment. **(A)** Changes in plant phenotype under drought treatment for 17 and 35 days. **(B–D)** Contents of glucose **(B)**, fructose **(C)**, and sucrose **(D)** in mature leaves. **(E)** Total SOD activity in mature leaves. Different letters indicate significant differences (one-way ANOVA, *p* < 0.05).

Under the drought treatment by irrigation depletion, the OE transgenic plants showed accelerated leaf senescence in the bottom leaves after 17 days of treatment. More severe symptoms of drought damage on cassava plants were observed, including middle leaf senescence, leaf shedding, and leaf dehydration (Figure 10A). Further analysis of sugar contents in the 17 days treated leaf samples showed significantly reduction in glucose, fructose, and sucrose contents in WT plants, but the changes in the OE transgenic plants were noticeable, showing significant reduction in glucose and fructose (Figure 10B–D). Before the drought treatment, the transgenic plants had lower levels of SOD activity than WT plants, approximately 10% less. SOD activities were increased in all plants after treatment and the WT plant showed the highest level (Figure 10E).

## Discussion

**FIGURE 11:**
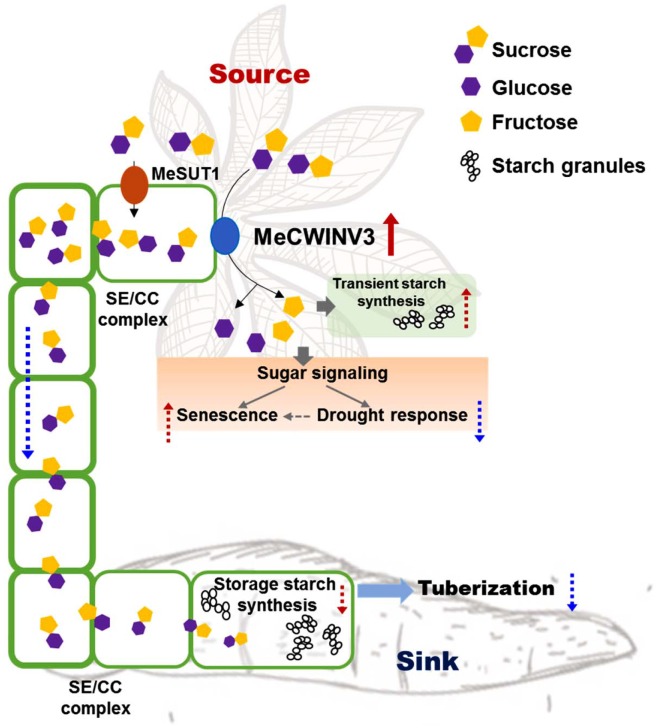
Schematic illustration of MeCWINV3 function on sucrose hydrolysis to regulate the sugar allocation from source to sink in vascular bundles. Normally, the photo-assimilates (sugars) from source cells are transported to root parenchyma cells via phloem loading mainly in the form of sucrose molecules for the use of starch biosynthesis to support storage root tuberization. In leaves, overexpression of MeCWIN3 promotes the sucrose hydrolysis (red up arrow), leading to increased transient starch accumulation, accelerated leaf senescence, and reduced drought tolerance. In stems, the rate of sucrose transportation is reduced. As a result, starchy storage root tuberization is repressed. Red up dash arrow indicates increased activity and blue down dash arrow indicates reduced activity.

Cassava produces storage roots as a major sink for storing starch derived from photoassimilates in the leaves (Alves, 2002). The storage process involves phloem loading and unloading of sucrose in the source and sink, respectively. Understanding the key factors in the pathway and its regulation enables us to modulate the process for better yield and improved nutrition in root crops. In this study, the function of MeCWINV3, a conserved protein of CWINVs that is highly expressed in vascular bundles, was studied for its role in carbohydrate partitioning from leaves to storage roots and plant growth in cassava. The results indicate that overexpression of *MeCWINV3* led to accelerated sucrose decomposition in cassava leaves, thus reducing the efflux of sucrose from source to sink and eventually causing early leaf senescence, reduced stress tolerance, and delayed storage root development in cassava (Figure 11). Our study provides new evidence of the importance of CWINVs in sugar homeostasis of source organs and development of higher plants (Ruan, 2014).

Many reports have shown that CWINVs play critical functions closely related to plant reproduction, affecting pollen germination, ovary activity, and fruit and seed development (Wang et al., 2008; Goetz et al., 2017; Ru et al., 2017; Wan et al., 2018). For example, down-regulated expression or knockout of CWINV genes reduced grain weight in maize (Miller and Chourey, 1992) and caused fruit and seed abortion in tomato (Jin et al., 2009; Zanor et al., 2009). In contrast, increased CWINV activity led to high oil content in cotton and high yield in Arabidopsis and maize (Von Schweinichen and Büttner, 2005; Li et al., 2013; Yang et al., 2017). Apical meristem-specific expression of CWINV also leads to accelerated flowering and an increase in seed yield by nearly 30% (Heyer et al., 2004). Suppressed expression of CWINV led to deficiency in carrot root development (Tang et al., 1999). Nevertheless, no detailed function in root crops like cassava has been reported and the way CWINV participate in sucrose hydrolysis to affect sugar allocation upon phloem loading has not been revealed in source organs. The high expression of *MeCWINV3* in cassava leaves indicates its role for sugar decomposition in apoplasts for sugar transport and intercellular sugar translocation among cells of vascular bundles (Figure 11). Its low expression in storage roots suggests fewer roles in sugar unloading in roots which might be conducted by other CWINV homologs such as MeCWINV1 (Yao et al., 2014). The expression of *MeCWINV3* had a nycthemeral rhythm in leaf, as INV LIN6 of tomato (Proels and Roitsch, 2009), indicating the key role of *MeCWINV3* in cassava source organs. In cassava, the apoplastic pathway is the major mode of sucrose transport, and therefore, MeCWINV3 might collaborate with sucrose transporters such as SWEETs and SUTs for sugar allocation (Eom et al., 2015). Our study has different results compared with those in Arabidopsis, maize or tomato (Von Schweinichen and Büttner, 2005; Li et al., 2013; Liu et al., 2016; Vallarino et al., 2017), in which overexpression of CWINV increased yield, owing to improved grain or fruit filling (Wang et al., 2008; Li et al., 2013). Altered sugar transport and impaired whole plant development had been reported in plants expressing the yeast CWINV due to disturbed assimilate partitioning (von Schaewen et al., 1990; Sonnewald et al., 1991; Heineke et al., 1994). Importantly, our results showed that constitutively overexpressing the *MeCWINV3* gene in cassava reduced biomass production, including plant height and storage root yield (Figure 11).

Our carbon isotope labeling data showed that sugar content, especially sucrose, significantly decreased in the stem phloem exudate of the OE transgenic cassava, but not in the labeled leaves. This suggests that excess MeCWINV3 activity in cassava decomposed more sucrose that is loaded into the phloem, thus reducing sugar transported from source to the sink. No changes in MeSUT1 expression was observed in the OE lines. As a result, sugar shortages were detected both in fibrous and storage roots, leading to reduced expression of starch biosynthetic genes such as *MeAPS*, *MeGBSSI*, and *MeSBEI*, possibly through a feedback mechanism of sugar signaling pathway (Yu et al., 2015). Consequently, the phenotypes of delayed storage root growth, reduced starch accumulation, and diminished yield were observed. There is no reduction of effective PSII quantum yield and non-photochemical quenching parameter in the leaves of transgenic OE lines, indicating less impact of their photosynthesis capacity. In cassava leaves, inhibition of the transport of assimilates in the phloem by petiole heat girdling showed a similar starch accumulation phenotype but different scenario of sugar contents in which reduced glucose and fructose were detected (Zhang Y. et al., 2015). Enhanced CWINV activity was detected during ovary-to-fruit transition in tomato and its LIN5 was localized to cell walls of sieve elements to facilitate phloem unloading and to generate a glucose signal for fruit set (Palmer et al., 2015). A recent study also proposed different roles of CWINV genes in seed development (Zhang et al., 2018). Therefore, this study reveals the important function of MeCWINV3 in maintaining cassava sugar homeostasis in source organs.

CWINV also influences plant stress response by altered sugar allocation. In tomato, fruit set under heat stress can be improved by increased CWINV activity, which suppresses ROS-independent cell death (Liu et al., 2016). Silencing CWINV inhibitor INVINH1 expression in tomato increased CWINV activity and enhanced chilling tolerance (Xu et al., 2017). In addition, overexpression of CWINV improved adaptation to drought stress of tomato (Albacete et al., 2015). CWINs may control the dynamic equilibrium of sugar metabolism, thus changing stress resistance of plants (Proels and Hueckelhoven, 2014). Cross-talk of CWINV with hormones has also been indicated; this affected crop yield by controlling sink activity (Rijavec et al., 2009; Albacete et al., 2014; French et al., 2014). The increased sink activity mediated by CWINV can lead to cytokinin-induced delay of senescence (Balibrea et al., 2004; Hwang et al., 2012). In contrast, the OE transgenic cassava showed an accelerated leaf senescence phenotype and reduced drought tolerance, indicating the importance of MeCWINV3 in plant growth and stress response (Figure 11). It is necessary to study further how CWINs influence both sugar metabolism and stress response in cassava.

Different CWINVs may fulfill variable functions, based on tissue specificity and enzymatic characteristics (Sturm, 1999; Lammens et al., 2009; Wan et al., 2018). Closely related defective CWINVs lacking sucrose degrading capacity may be involved in sequestering CWINV inhibitors (Le Roy et al., 2013). They lack the typical D/K or D/R couple that to bind sucrose as a substrate. MeCWINV3 contains the D/K couple and all other typical characteristics of a CWINV (Lammens et al., 2008; Le Roy et al., 2013), in line with its proposed function in sucrose hydrolysis.

## Conclusion

In conclusion, cassava *MeCWINV3* is an important CWINV gene that functions in vascular bundles for sugar allocation, especially in source leaves. Its constitutive overexpression in cassava reduced biomass production, accelerated leaf senescence, and delayed storage root growth due to the inhibition of sugar transport from leaves to storage roots. The low level of sugars in storage roots affects starch biosynthesis and accumulation. Our study reveals the critical role of MeCWINV3 in regulating sucrose translocation and homeostasis in cassava, thus affecting source capacity and sink unloading of cassava.

## Author Contributions

WY and XW performed most of the experiments, analyzed the data, and draft the manuscript. YL, GL, ZC, TJ, and QM conducted partial experiments. LL and PZ conceived and designed the study, analyzed the data, and revised the manuscript with input from other authors. All authors discussed the results and approved the final manuscript.

## Conflict of Interest Statement

The authors declare that the research was conducted in the absence of any commercial or financial relationships that could be construed as a potential conflict of interest.

## Footnotes

1 https://phytozome.jgi.doe.gov/pz/portal.html#!info?alias=Org_Mesculenta
