# Supplementary material for: Cell Wall Invertase 3 Affects Cassava Productivity via Regulating Sugar Allocation From Source to Sink
Source: Front Plant Sci. 2019 Apr 30;10:541. doi: 10.3389/fpls.2019.00541 (PMC6503109; doi:10.3389/fpls.2019.00541)
Supplement: Supplementary file 1 [file Data_Sheet_1.PDF]

Supplementary Material

1 Supplementary Table

Table S1 Primers used in this research for RT-PCR analyses

Gene locus	Names of primer pair	Sequence (5'-3')
Manes.03G049200	*MeCWINV1*-RT F	GGTGTTGCAAAGGACGATTT
	MeCWINV1-RT R	TCCACACCATTTGTGCTGTT
Manes.08G027200	*MeCWINV2*-RT F	GCTGTTCAGGCTGATGTTGA
	MeCWINV2-RT R	CATGTTTGGTTGGTGCTTTG
Manes.11G025400	*MeCWINV3*-RT F	TCCCTGGTGTCACAGCATC
	MeCWINV3-RT R	TAACTGAGCCACCCTTTCG
Manes.04G140500	*MeCWINV4*-RT F	GCCAATCCAAGAAATCGAAA
	MeCWINV4-RT R	GGCAAGCACCGTAATTTTGT
Manes.08G027300	*MeCWINV5*-RT F	CATCCTTTGCTGGGTTTGTT
	MeCWINV5-RT R	CTCCAAGCACCGAGTTTCTC
Manes.09G053500	*MeCWINV6*-RT F	TCTGCAACAATGGCGTAGAG
	MeCWINV6-RT R	CGTTGCTCCATTAGGGTTGT
Manes.13G058900	*MeAPS*-RT F	ACCTCTGCAAAGCCTCTCAT
	MeAPS-RT R	ACTAGCGTCAGGGTCAAGAC
Manes.02G001000	*MeGBSSI*-RT F	CGTGAAGGGAAGGAAAATCA
	MeGBSSI-RT R	TGTGGCATCGTAGTGGATGT
Manes.05G133800	*MeSBEI*-RT F	GCTCGCACTTGTGTGGTTTA
	MeSBEI-RT R	CATCGGCAATCAAAGAAGGT
Manes.03G114300	*MeNAC2*-RT F	CGCTAGACCTGCAGAGCTAT
	MeNAC2-RT R	CTGAAAGCTGGTATGGTGGC
Manes.09G185300	*MeNAC83*-RT F	TCAGCACCAGGGAAGCTAAA
	MeNAC83-RT R	TGAGGGGAATTGCAGACAGT
Manes.02G108300	*SAG12*-RT F	GATCATGGTGTTGCTGCTGT
	SAG12-RT R	*CAGTGGGGTAAGATGCTTGC*
Manes.08G069100	*Osl*-RT F	GATTGCCCTCATTACTGGCG
	Osl-RT R	TCCAAGCCTTCCAAATGCAC
Manes.09G114900	*Osh*-RT F	CCAAAAGGCTCCTCTCCAGA
	Osh-RT R	GGAGGGGCTGAGAAACTCTT
Manes.18G099400	*MeSUT1*-RT F	ATTCCTTTTGCAATGGCATC
	MeSUT1-RT R	TTTGGCTGAGGGGATATCAG
Manes.05G099000	*MeSUT2-RT* F	CAAAATGGCCTTGAGCTTTC
	MeSUT2-RT R	GCTCCAGGTCCATCACCTAA
Manes.05G186600	*MeSUT4-RT* F	TGTAGAGCGCTTCTTGCTGA
	MeSUT4-RT R	GCAGACTTGAGATTGGCACA
Manes.12G150600	*β-Actin* F	TGATGAGTCTGGTCCATCCA
	β-Actin R	CCTCCTACGACCCAATCTCA
Manes.11G025400	*MeCWINV3*-p1301s F	TCCCCCGGGATGGACATCTTCTCCATGAAG
	MeCWINV3-p1301s R	ACGCGTCGACTCAGTTGATGTGAGCCTTCT
Manes.11G025400	*MeCWINV3*-YFP F	GTAGTCGACATGGACATCTTCTCCATGAA
	MeCWINV3-YFP R	ATTCCCGGGGTTGATGTGAGCCTTCTTCA

2 Supplementary Figures

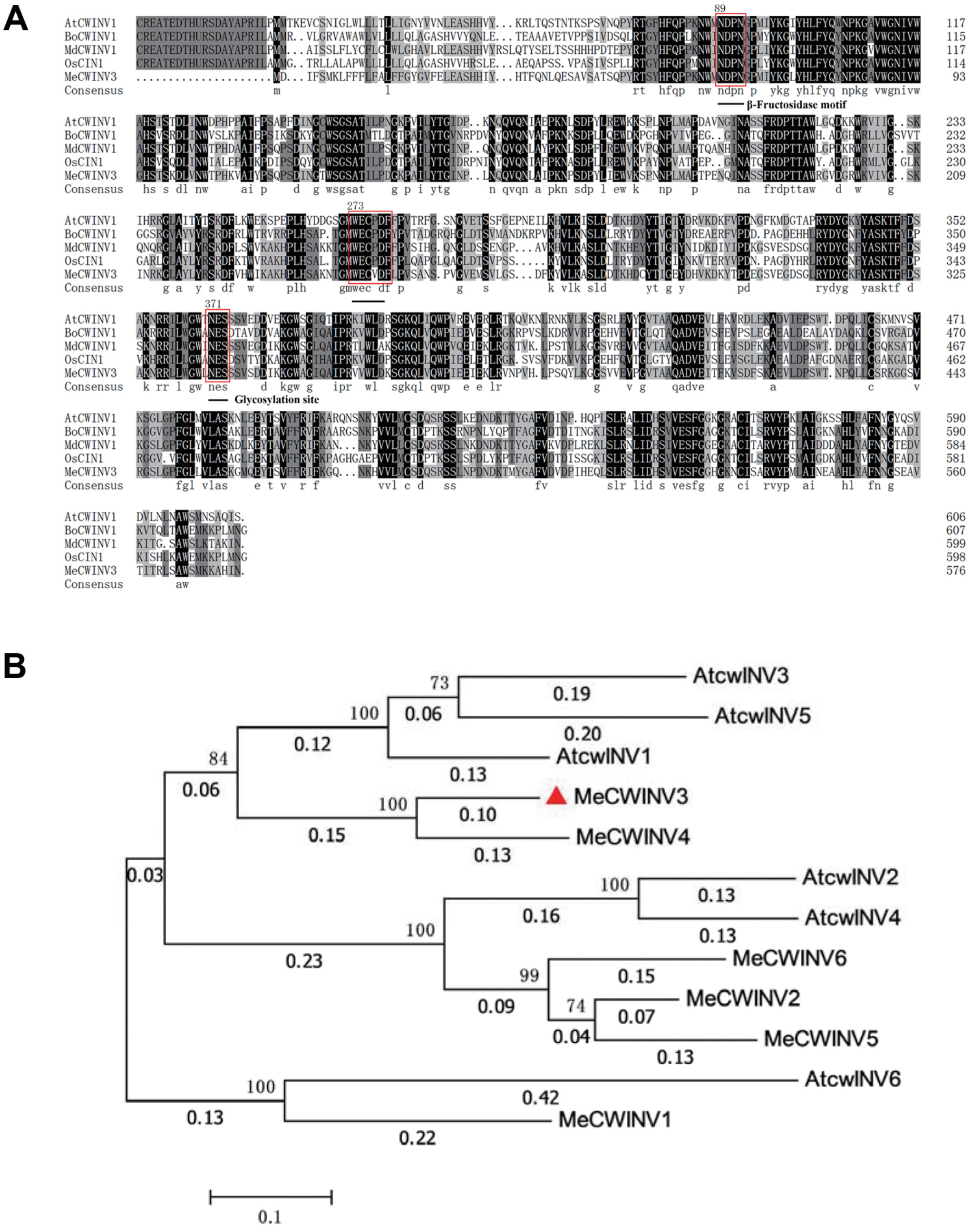

Figure S1 Alignment of amino acid sequences and phylogenetic tree of CWINVs. (A) Multiple alignments of amino acid sequences of cassava MeCWINV3 (GenBank accession No. JN801147.1), Arabidopsis AtcwINV1 (GenBank accession No. AT3G13790), *Bambusa oldhamii* BoCWINV1 (GenBank accession No. DQ267823.1), *Malus domestica* MdCWINV1 (GenBank accession No. JX459588.1) and *Oryza sativa* OsCIN1 (GenBank accession No. AB073749.1). The boxed nucleotides represent the conserved motifs for three conserved sequence domains - NDPNG (β-fructosidase motif), RDP, and WECP(V)D. (B) Maximum likelihood tree of CWINVs from cassava and Arabidopsis.

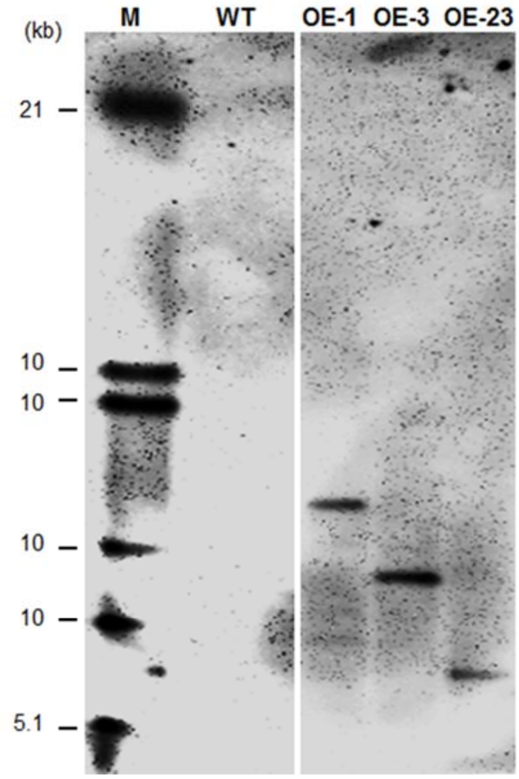

Figure S2 Integration patterns of T-DNA in *MeCWINV3*-overexpressed cassava plants analyzed by Southern blotting. M, DIG-labeled molecular marker; WT, wild-type control; OE lines, *MeCWINV3*-overexpressed transgenic lines.

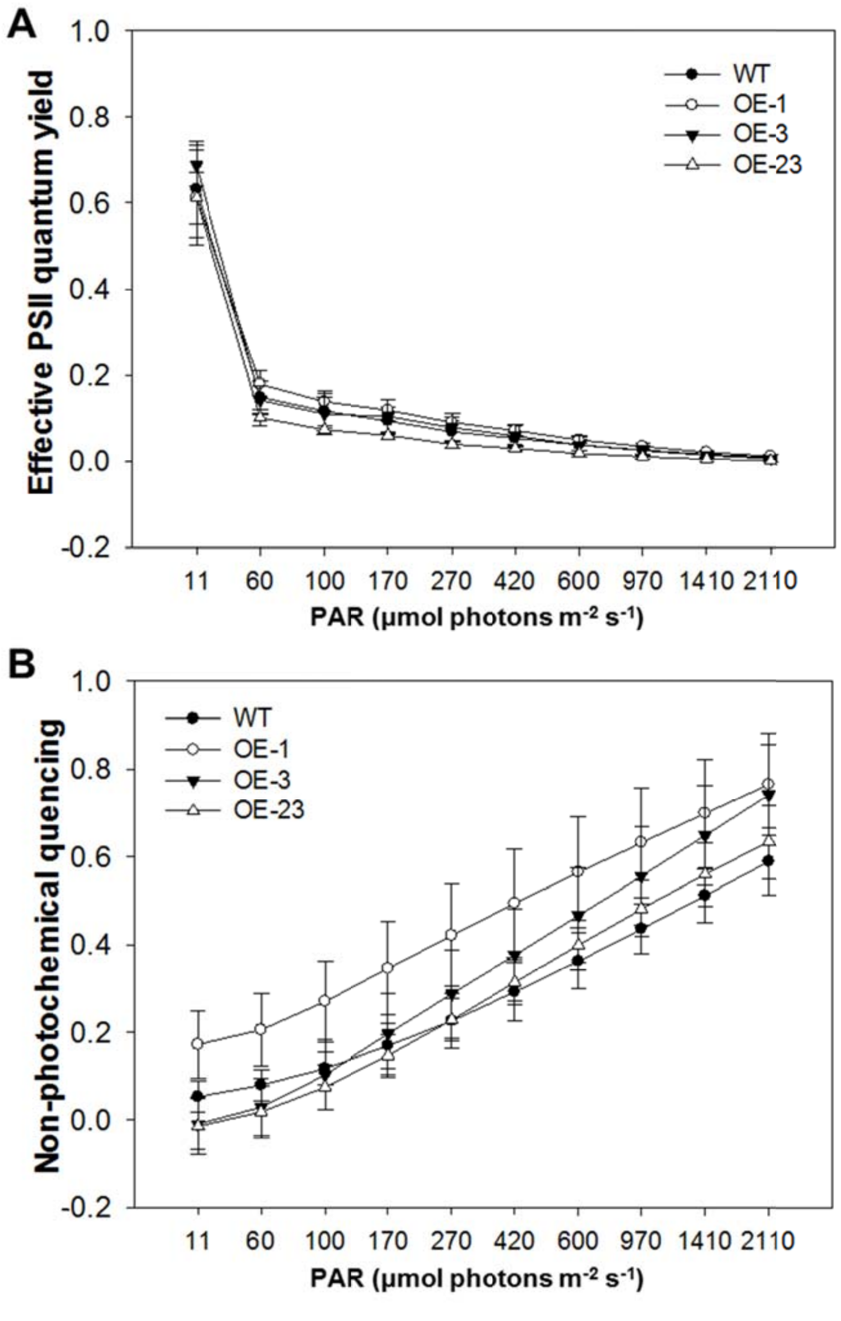

Figure S3 Measurement of light intensity response curves of wild type (WT) and the *MeCWINV3* overexpressing lines OE-1, OE-2 and OE-3. (A) Effective PSII quantum yield. (B) Non-photochemical quenching parameter. Leaves of plantlets from in vitro cultures were used and fluorescence measurements were performed using a portable chlorophyll fluorometer PAM2000 (Walz, Germany).

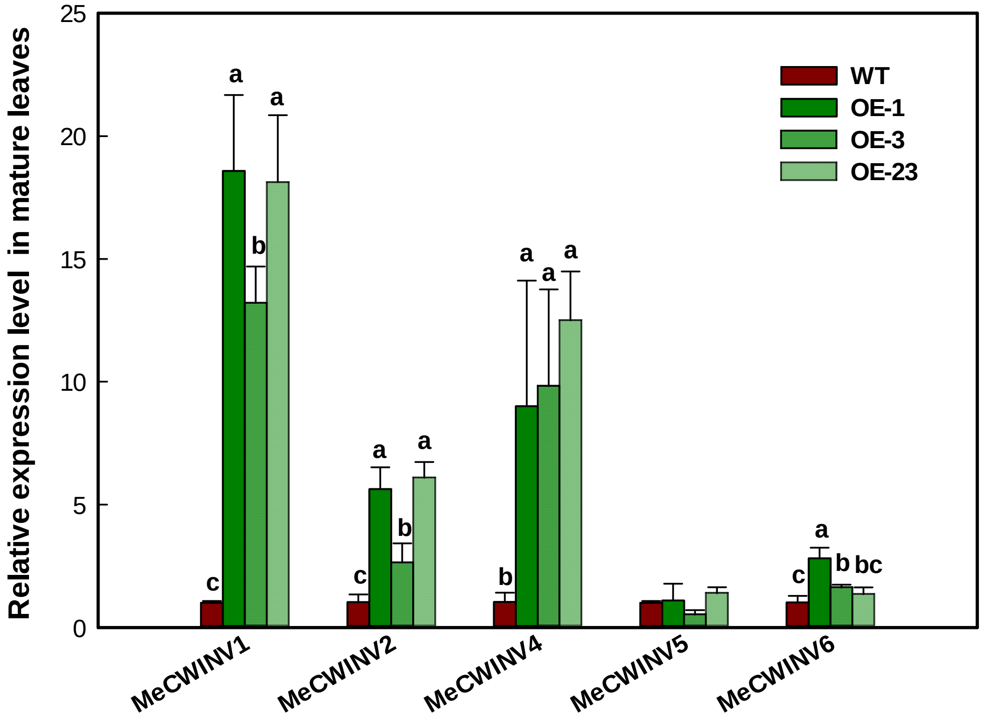

Figure S4 Relative expressions of CWINV isoforms in the leaves of *MeCWINV3* overexpressing lines (OE-1, OE-2 and OE-3) in comparison with the wild type (WT) by the Real-time RT-PCR. Different letters indicate significant differences (one-way ANOVA, $p < 0.05$).

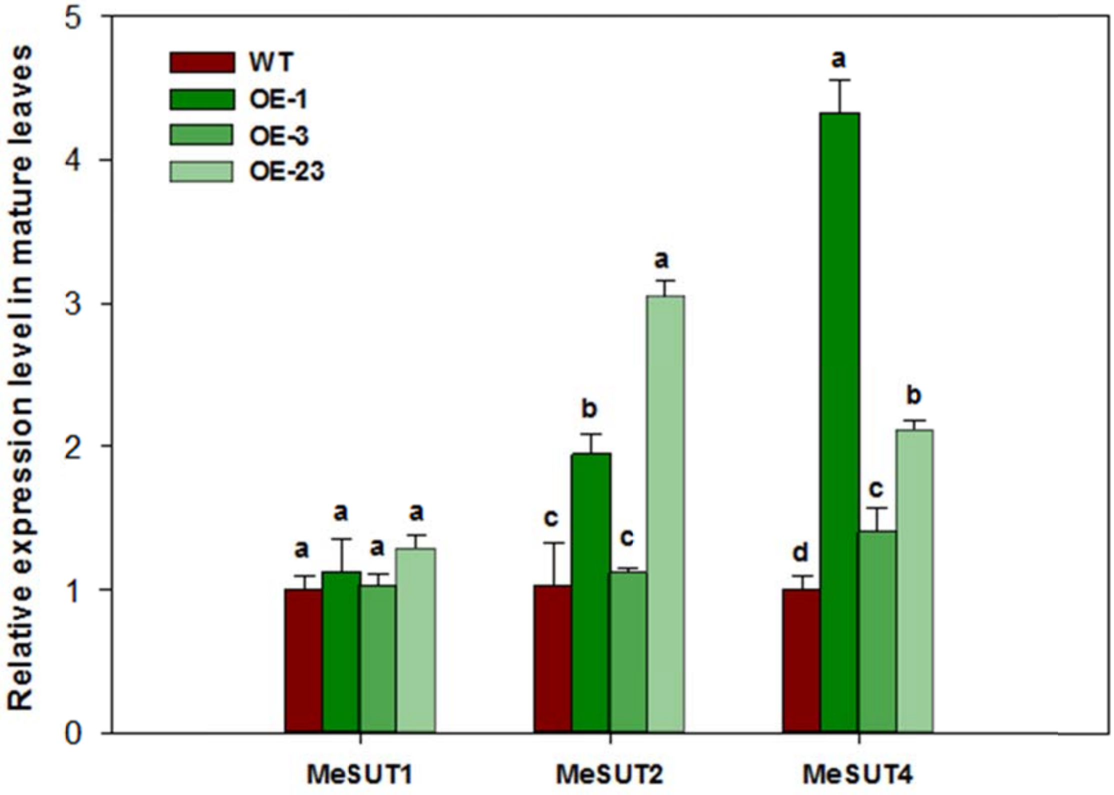

Figure S5 Relative expressions of sucrose transporter genes *MeSUT1*, *MeSUT2* and *MeSUT4* in the leaves of *MeCWINV3* overexpressing lines (OE-1, OE-2 and OE-3) in comparison with the wild type (WT) by the Real-time RT-PCR. Different letters indicate significant differences (one-way ANOVA, $p < 0.05$).

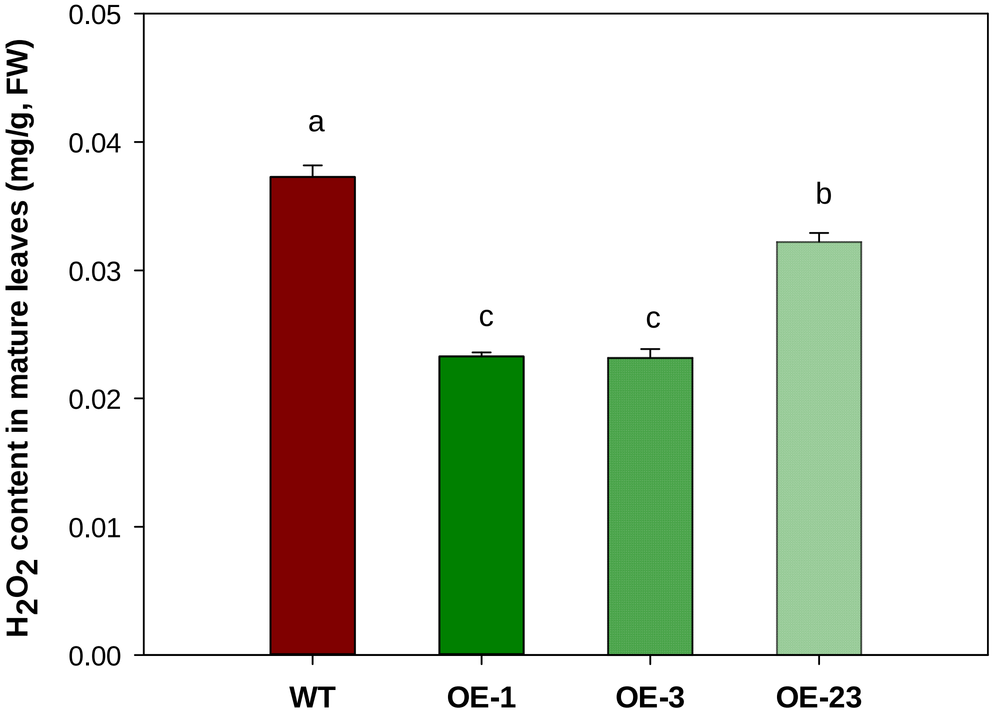

Figure S6 H_2O_2 content in the leaves of *MeCWINV3* overexpressing lines (OE-1, OE-2 and OE-3) in comparison with the wild type (WT) under the normal growth conditions. Different letters indicate significant differences (one-way ANOVA, $p < 0.05$).
